# Development and validation of the AI dependence scale for Chinese undergraduates and a preliminary exploration

**DOI:** 10.3389/fpsyg.2025.1725393

**Published:** 2026-01-19

**Authors:** Houyu Wu, Haiyang Ni, Wenfu Luo, Tenglong Wu

**Affiliations:** 1 Personnel Department, Neijiang Normal University, Neijiang, China; 2 Faculty of Education, Languages, Psychology and Music, SEGi University, Nilai, Malaysia; 3 Graduate School of Business, Nilai University, Selangor, Malaysia; 4 Basic Teaching Department, Xinjiang University of Politics and Law, Tumxuk, China; 5 College of Pharmacy, Zhejiang Pharmaceutical University, Ningbo, China

**Keywords:** AI dependence, Chinese undergraduates, higher education, psychometrics, scale development

## Abstract

**Introduction:**

With the proliferation of generative artificial intelligence (AI) in higher education, student overreliance has become a growing concern, potentially undermining critical thinking and autonomous learning. To address the lack of a comprehensive measurement tool, this study developed and validated the AI Dependence Scale (AIDep-22), a new instrument designed to assess this phenomenon across four hypothesized dimensions: emotional dependence, functional dependence, cognitive dependence, and loss of control.

**Methods:**

The scale was constructed following a rigorous two-stage process, beginning with item generation and refinement through expert reviews and cognitive interviews, followed by psychometric evaluation with two independent samples of Chinese university students (*N* = 400 each).

**Results:**

An exploratory factor analysis (EFA) supported the four-factor structure, which was subsequently confirmed by a confirmatory factor analysis (CFA) on the second sample. The final 22-item scale demonstrated excellent internal consistency (Cronbach's alpha = 0.87), strong convergent and discriminant validity, and robust criterion-related validity. Preliminary analyses also identified key demographic risk factors, revealing that male students, upper-year students, those in applied majors, and more frequent AI users reported significantly higher dependence.

**Discussion:**

This study contributes a reliable and valid diagnostic tool that enables educators and researchers to identify and support students at risk, and to design targeted interventions that promote a more balanced human-AI relationship in higher education.

## 1 Introduction

The proliferation of artificial intelligence (AI) in higher education presents both profound opportunities and significant challenges (Valcea et al., 2024). As university students increasingly leverage tools such as ChatGPT to streamline assignments and problem-solving, gains in efficiency are accompanied by growing concerns regarding overreliance (Essien et al., 2024; Li et al., 2024). This overreliance manifests as an uncritical dependence on AI-generated outputs, potentially outsourcing higher-order cognitive processes and reflective judgment (Gonsalves, 2024). Empirical evidence links sustained reliance on AI chatbots to weakened critical thinking and problem-solving skills; specifically, prolonged AI use has been associated with measurable declines in independent analytical abilities (Essel et al., 2024). A recent systematic review reinforces this view, suggesting that while AI enhances productivity via rapid content generation, excessive dependence erodes originality and independent thought, ultimately stunting long-term intellectual growth by cultivating passivity (Zhai et al., 2024). Collectively, these findings suggest a shift wherein AI tools devolve from cognitive supports into shortcuts that weaken self-regulation and autonomy. This pattern mirrors earlier trajectories of internet and smartphone dependence, where chronic use altered cognitive habits and diminished intrinsic motivation.

The broader research on technology dependence provides a conceptual foundation for this phenomenon. Specifically, research on social media addiction frames dependence as a multi-dimensional construct encompassing emotional, cognitive, and behavioral domains. For instance, the Bergen Facebook Addiction Scale operationalizes dependence via components such as salience, mood modification, tolerance, withdrawal, conflict, and relapse (Andreassen et al., 2012). This model emphasizes impaired control and negative consequences over usage frequency alone, providing a robust framework for assessing digital behavioral addictions (Griffiths, 2005). Similarly, the Media and Technology Usage and Attitudes Scale highlights emotional reactivity, such as heightened anxiety or irritability during periods of non-use, alongside behavioral dysregulation, underscoring how affective responses perpetuate cycles of dependence (Rosen et al., 2013). These scales demonstrate that technology dependence is not a static state but a dynamic interplay of psychological factors, in which initial voluntary engagement escalates into involuntary, even compulsive, use with adverse outcomes.

Studies on smartphone addiction extend these insights to ubiquitous devices, establishing parallels for AI integration in the academic environment. Specifically, investigations identify preoccupation and disengagement difficulties as central features of problematic use among university students (Abuhamdah and Naser, 2023). These behaviors are linked to functional costs spanning both cognitive and physical domains, manifesting as reduced cognitive flexibility evidenced by impaired task-switching, alongside general physical discomfort resulting from prolonged and static device engagement (Rodríguez-Brito et al., 2022; Inal and Serel Arslan, 2021). Synthesizing these findings, technology dependence in higher education emerges not as a monolithic addiction metric but as a complex interplay of instrumental reliance and psychological attachment, mirroring the structural complexity observed in social media addiction research (Ross and Campbell, 2021; Serenko and Turel, 2020). This cumulative evidence critically informs the analysis of AI dependence, suggesting that pervasive technologies erode autonomy through similarly repeated and reinforcing interactions.

Based on this background, this study defines AI dependence as a persistent pattern of excessive reliance on AI tools for academic purposes, wherein emotional regulation, instrumental academic functioning, higher-order cognition, and behavioral self-control become increasingly contingent on AI systems. This conceptualization integrates three theoretical streams. First, behavioral addiction theory underscores impaired control and continued use despite negative consequences, explaining how initial experimentation evolves into habitual patterns that prioritize short-term relief over skill development (Griffiths, 2005; Serenko and Turel, 2020). Second, technology acceptance model (TAM) suggests that while perceived usefulness drives adoption, it may inadvertently foster overreliance when students internalize a sense of inadequacy without AI support, generating a feedback loop in which the system is perceived as indispensable (Davis et al., 1989; Parveen et al., 2024; Shahzad et al., 2024). Third, cognitive offloading research posits that delegating demanding cognitive tasks to external tools, though momentarily efficient, becomes maladaptive when it systematically displaces internal processing and memory consolidation, ultimately resulting in skill atrophy (Grinschgl et al., 2021; Gilbert, 2024; Wahn et al., 2023).

**Figure 1:**
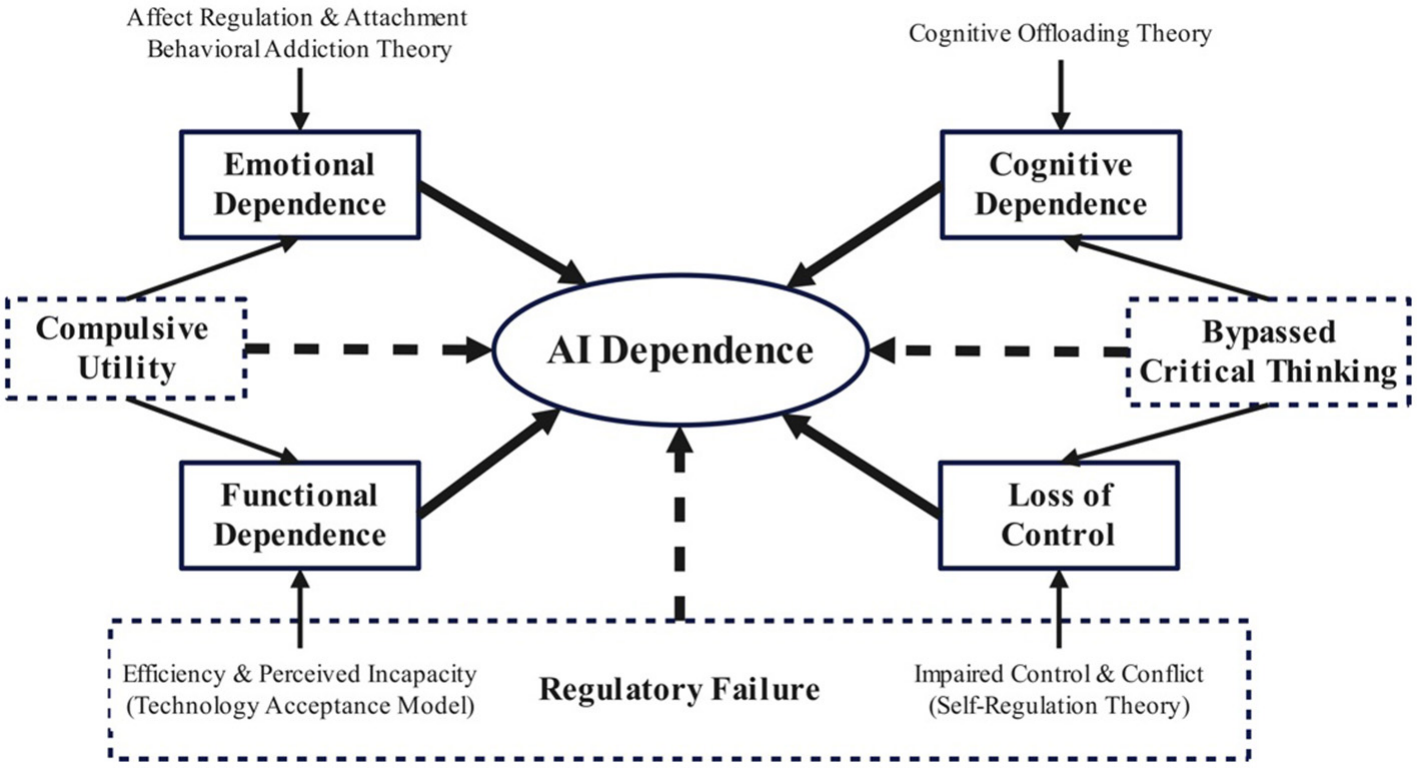
The conceptual framework of this study.

In line with these perspectives, this study conceptualizes AI dependence as a multidimensional construct comprising four interrelated dimensions: emotional dependence pertains to using AI for affect regulation and experiencing negative emotions when unavailable; functional dependence reflects overreliance on AI for task execution and productivity; cognitive dependence involves the delegation of higher-order thinking and decision-making to AI; loss of control signifies difficulty regulating AI use despite intentions and perceived costs. These dimensions map onto affective regulation, instrumental reliance, cognitive delegation, and behavioral self-regulation respectively, thereby ensuring a holistic framework that addresses the multifaceted nature of AI dependence in higher education. The conceptual model is illustrated in Figure 1.

While conceptual parallels with other digital technologies exist, AI systems possess unique affordances such as real-time personalization and adaptive learning, which limit direct extrapolation from existing scales. Early instruments, such as the Dependence on AI Scale, provide initial insights but adopt unidimensional structures with limited item pools, making it challenging to differentiate whether elevated scores arise from emotional comfort, functional convenience, or impaired control (Morales-García et al., 2024). Similarly, the Conversational AI Dependence Scale addresses chatbot-based problem-solving but mirrors generic smartphone addiction templates, lacking conceptual nuance specific to academic cognitive demands (Chen et al., 2025). Other measures, like the Problematic ChatGPT Use Scale, emphasize addiction-like symptoms such as craving and interference, but predominantly treat AI dependence as a single latent construct, rarely validating nuanced multidimensional models (Maral et al., 2025; Zhang et al., 2025). Although behaviorally oriented measures have identified distinct usage patterns, they often avoid framing these as psychological dependence, thereby overlooking underlying emotional vulnerability or cognitive inertia mechanisms (Hou et al., 2025).

These contributions highlight AI dependence as a critical issue and substantial gaps persist. Conceptually, most scales transplant addiction markers from other technologies without systematically integrating theories of technology acceptance, cognitive offloading, and self-regulation into a unified framework. Psychometrically, the dominance of unidimensional models limits the capacity to distinguish whether problematic use is primarily emotional, functional, cognitive, or regulatory. This lack of dimensional clarity complicates the design of targeted interventions, as strategies for emotional reassurance differ fundamentally from those addressing cognitive inertia or self-control failures.

Consequently, there is a critical need for a theoretically grounded, multidimensional scale operationalizing AI dependence across these facets. To address this gap, this study developed and validated the AI Dependence Scale (AIDep-22) for university students, grounded in behavioral addiction theory, technology acceptance research, and cognitive offloading models. This instrument conceptualizes students' AI dependence as excessive academic reliance manifesting through emotional dependence, functional dependence, cognitive dependence, and loss of control. The following subsections detail each dimension.

### 1.1 Emotional dependence

Emotional dependence captures the extent to which students resort to AI, not merely for information but as a primary source of affective regulation. While previous discussion established the link between technology and mood modification, AI dependence introduces a unique psychological dynamic distinct from social media. Unlike the interpersonal validation sought on social media, the attachment to AI stems from its capacity to offer instant and non-judgmental reassurance during moments of academic vulnerability (Hu et al., 2023). This creates a “safe space” mechanism where the tool mitigates the intrinsic anxiety of learning processes such as uncertainty and self-doubt (Parmaksiz, 2022). Consequently, the absence of AI triggers specific negative affective reactions including anxiety, frustration, and insecurity which reflect a deeper emotional fragility rather than simple operational inconvenience (Zhang et al., 2024).

This phenomenon deepens the understanding of technology-mediated attachment. While general constructs like nomophobia describe anxiety arising from disconnection (Wang et al., 2021; Wen et al., 2023), AI emotional dependence suggests a more targeted reliance on the tool's perceived “omniscience” to manage academic stress. Related longitudinal evidence on fear of missing out (FoMO) shows that unmet psychological needs and FoMO-driven social media use can reinforce cycles of emotional vulnerability and compulsive checking (Groenestein et al., 2025), offering a useful analog for understanding how AI-based reassurance may similarly entrench avoidance of academic discomfort. Instruments such as the Media and Technology Usage and Attitudes Scale highlight emotional reactivity to non-use (Rosen et al., 2013), but current AI research often overlooks how this reactivity specifically erodes academic resilience (Goldfine et al., 2022). The critical danger lies in the shift from using technology to facilitate learning to employing it as a “psychological crutch”, for escaping the emotional discomfort inherent in intellectual challenge (Dou et al., 2024; Hamamura et al., 2023).

In the development of the AIDep-22, this dimension focuses on the maladaptive intersection of affect and utility. Items are designed to measure the intensity of unease or tension experienced without AI access and the degree of psychological comfort derived from human-AI interaction. This operational approach aligns with the withdrawal component of behavioral addiction, but specifically contextualizes it within the emotional landscape of higher education where the AI functions as an external regulator of academic self-esteem.

### 1.2 Functional dependence

Functional dependence describes the mechanism by which AI shifts from a practical support tool to a compulsive necessity, making students unable to complete tasks independently in its absence. While acceptance-related models posit usefulness as a driver of adoption, a critical analysis reveals a divergent trajectory in higher education: unlike workplace tools where efficiency is the ultimate metric, academic learning requires “desirable difficulties” to foster competence (Bearman and Ajjawi, 2023). Functional dependence arises when the efficiency gains from AI, such as rapid drafting or synthesis, induce a state of perceived incapacity where students feel unable to execute these tasks unaided (Parveen et al., 2024). This reflects a transition from voluntary adoption to a compulsive reliance where the tool is no longer an extension of ability but a substitute for it.

This dimension critically reinterprets high-frequency usage of AI tools. While system-usage research typically interprets extensive engagement as positive integration, in the context of AI, such metrics may mask underlying skill degradation (Zhai et al., 2024). Recent studies indicate that while AI reduces cognitive load, unregulated instrumental reliance creates a dependency trap (Abbas et al., 2024; Lo et al., 2024). Students may maintain high productivity specifically by outsourcing the procedural steps of information gathering and organizing to the system (Zhang et al., 2024; Liu, 2025). Consequently, the demarcation between effective utilization and functional dependence lies not in usage frequency, but in the psychological erosion of self-efficacy, where the user perceives an inability to execute tasks independently once the tool is withdrawn (Chan and Hu, 2023).

The AIDep-22 operationalizes this dimension by targeting the student's self-perception of efficacy without AI. Items capture the specific sensation of task paralysis or inadequacy when AI assistance is removed rather than merely quantifying usage frequency.

### 1.3 Cognitive dependence

Cognitive dependence examines the structural transformation of thinking processes under the influence of persistent AI scaffolding. Extending the cognitive offloading framework introduced previously, this dimension highlights the risk of “cognitive inertia” where the human tendency to conserve mental effort is amplified by the availability of AI. While offloading can strategically free up resources for complex tasks, cognitive dependence involves the habitual delegation of higher-order processes including analysis, argumentation, and critical judgment (Grinschgl et al., 2021; Wahn et al., 2023). The core issue is not the use of AI for assistance but the systematic bypassing of the constructive friction required for deep learning and memory consolidation during use (Gong and Yang, 2024; Gerlich, 2025).

The critical review of AI literature suggests that this dependence fosters an illusion of competence. Students may produce high-quality outputs with AI and mistakenly attribute this performance to their own ability, thereby masking a decline in actual critical thinking skills (Gonsalves, 2024). This aligns with warnings from cognitive psychology that when external agents consistently generate ideas or structures, the user's internal schemas for these tasks weaken through disuse (Jose et al., 2025). The danger is a gradual surrender of intellectual autonomy where the student prefers the algorithmic judgment of the AI over their own reasoning.

The AIDep-22 distinguishes this dimension by focusing on the displacement of cognitive effort. Items assess behaviors such as soliciting answers without prior reflection, a preference for AI-generated logic over personal critique, and a perceived deterioration in independent problem-solving skills. This operationalization links the theoretical concerns of cognitive atrophy directly to measurable student behaviors and emphasizes the loss of intellectual agency rather than just the act of using the tool.

### 1.4 Loss of control

Loss of control represents the regulatory failure that cements the transition from heavy use to problematic dependence. While the previous dimensions explain the motivations for reliance including emotional comfort, functional need, or cognitive ease, this dimension captures the inability to control the engagement of using AI. Critically, this dimension disentangles “high engagement” from “addiction”. Students may use AI intensively for a specific project without being dependent. However, loss of control is characterized by the breakdown of volitional inhibition where usage persists despite conflicts with other academic duties or the recognition of diminishing returns (Griffiths, 2005; Andreassen et al., 2012).

Existing measures often conflate high frequency of use with loss of control, but research on digital addiction suggests that the defining feature is the failure of self-regulation strategies (Hamamura et al., 2023; Throuvala et al., 2021). In the context of AI usage in higher education, this manifests as compulsive “prompt engineering” or an inability to stop refining outputs even when the marginal gain is negligible (Ivanov et al., 2024). Emerging AI scales report symptoms like craving and compulsive checking but often fail to isolate this regulatory deficit from simple functional reliance (Morales-García et al., 2024; Chen et al., 2025). By treating loss of control as a distinct dimension, this approach clarifies that the pathology lies in the students' diminished agency over their interaction with the system.

In the AIDep-22, loss of control is operationalized through items that reflect unsuccessful attempts to reduce usage, the dominance of AI interaction over other essential activities, and a subjective sense of being unable to limit engagement. This specific focus ensures that the scale identifies students who have lost the capacity to strategically deploy the tool and instead exhibit the compulsive usage patterns characteristic of behavioral addiction.

## 2 Method

The development and validation of the AIDep-22 adhered to the classical paradigm for scale construction articulated by (Hinkin 1998) and (DeVellis and Thorpe 2021). The research was structured in two main stages. The initial stage involved three foundational phases: (1) item generation, (2) content validation, and (3) pilot testing. Following this, a second stage was conducted, which consisted of a large-sample psychometric evaluation. This evaluation was designed to establish the scale's factor structure through exploratory and confirmatory factor analyses (EFA and CFA), assess its internal consistency, and confirm its convergent and discriminant validity. Furthermore, the scale's criterion validity was examined by assessing its associations with academic self-efficacy and AI usage behaviors. As a final step, an initial application of the instrument involved an exploratory analysis of AI dependence levels across different student demographic groups.

### 2.1 Item generation

Guided by the proposed four-dimensional framework, an initial pool of 26 self-report items was generated to measure AI dependence. The formulation of these items was grounded in established instruments that measure technology dependence and acceptance. Specifically, items for the emotional dependence and loss of control dimensions were adapted from existing mobile phone addiction scales, targeting concepts such as anxiety during non-use and difficulty in regulating usage (Dou et al., 2024; Hamamura et al., 2023). Items from the Media and Technology Usage and Attitudes Scale were also consulted for these dimensions (Goldfine et al., 2022). For example, smartphone items that assess feeling nervous or frustrated when unable to use the device were recontextualized into the academic AI setting, yielding items such as Item 3 (“I would feel anxious and frustrated if I couldn't use AI for my studies”), while items capturing unsuccessful attempts to reduce smartphone use informed loss-of-control items such as Item 21 (“I have tried to reduce my use of AI, but I have never succeeded for long”). To assess functional dependence, statements from Technology Acceptance Model questionnaires were modified; for example, items originally measuring perceived usefulness were reframed to capture dependence on AI for task completion and efficiency (Davis et al., 1989), resulting in items like Item 7 (“I delegate almost all my academic tasks to AI because it performs them faster and better than I do”). Finally, items for the cognitive dependence dimension were developed based on the concept of cognitive offloading, aiming to measure the delegation of thinking tasks to AI (Throuvala et al., 2021).

All items were constructed as first-person statements situated within an academic context. Responses were captured on a five-point Likert scale, ranging from 1 (strongly disagree) to 5 (strongly agree), with higher scores indicating a greater level of dependence. To facilitate interpretation, mean scores between 1.00 and 2.49 were classified as low dependence, scores between 2.50 and 3.49 as moderate dependence, and scores of 3.50 and above as high dependence. As detailed in Table 1, this initial pool comprised six items for emotional dependence, seven for functional dependence, six for cognitive dependence, and seven for loss of control. The item content was intentionally designed to be clear and relatable to common student experiences, avoiding overly technical language. To mitigate acquiescence bias, the item pool included a mix of both negatively phrased items reflecting dependence symptoms (e.g., feeling anxious) and positively phrased items indicating reliance (e.g., feeling supported).

**Table 1:** Initial item pool (26 items) and proposed dimensions of the AIDep-22.

**Dimension**	**Item ID**	**Item description**
Emotional dependence	Item1	I feel uneasy or insecure without the help of AI.
	Item2	Using AI to assist my studies makes me feel calm and relaxed.
	Item3	I would feel anxious and frustrated if I couldn't use AI for my studies.
	Item4	I've grown accustomed to completing academic tasks with AI; being without it would make me feel unsettled.
	Item5	Whenever I encounter a difficult problem, my first instinct is to hope AI can help me; otherwise, I feel tense.
	Item6	Interacting with AI (e.g., via chat prompts) is a pleasant experience that makes me feel supported.
Functional dependence	Item7	I delegate almost all my academic tasks to AI because it performs them faster and better than I do.
	Item8	Without the help of AI, I find it difficult to complete my assignments efficiently.
	Item9	I rely on AI's functions (e.g., providing answers, writing suggestions) to improve my learning efficiency.
	Item10	I can no longer manage complex academic tasks without the assistance of AI.
	Item11	Compared to doing it myself, I prefer to let AI gather information or organize content for me.
	Item12	In my studies, I excessively rely on the ready-made answers or solutions provided by AI.
	Item13	I feel inadequate in my studies without the support of AI.
Cognitive dependence	Item14	I often ask AI for answers directly without thinking for myself first.
	Item15	After using AI, my ability to independently analyze and solve problems has declined.
	Item16	When faced with an academic challenge, I rely on AI instead of attempting to think it through on my own.
	Item17	After long-term use of AI, I find I've become mentally lazy and always want AI to think for me.
	Item18	I am accustomed to letting AI make judgments and decisions for me rather than fully trusting my own judgment.
	Item19	In my studies, I tend to delegate tasks that require deep thinking to AI.
Loss of control	Item20	Even when it's not necessary, I find myself involuntarily using AI to assist with my studies.
	Item21	I have tried to reduce my use of AI, but I have never succeeded for long.
	Item22	Once I start using AI, I often find it difficult to stop, even after spending a lot of time on it.
	Item23	Sometimes I know I shouldn't rely on AI excessively, but I just can't control myself.
	Item24	I have almost no control over the frequency and duration of my AI use.
	Item25	I could complete tasks without AI, but I just can't resist using it.
	Item26	If I don't use AI for a period of time, I feel a strong urge to use it, sometimes even feeling irritable.

### 2.2 Expert review for content validity

To establish content validity, the initial 26-item draft of the AIDep-22 was submitted for evaluation by an expert panel. The panel comprised five members with relevant expertise: two professors of educational technology, one professor of educational measurement and evaluation, and two associate professors specializing in technology addiction. All experts were affiliated with public universities in Southwest China and had extensive experience in either scale development or empirical research on technology-related behaviors in higher education. Each expert independently rated all items on three core criteria: clarity, relevance, and necessity. A four-point rating scale, from 1 (not at all) to 4 (very much), was utilized for each criterion. Based on the relevance ratings, item-level content validity index (I-CVI) was calculated as the proportion of experts assigning scores of three or four. A scale-level CVI was then derived as the average of all I-CVI values. Following common recommendations, items with I-CVI values below 0.8 were flagged as candidates for revision or deletion. Necessity ratings were additionally used to compute content validity ratios (CVR) to determine whether an item was considered essential by a majority of experts, further informing decisions about item retention.

Following a synthesis of the expert feedback, a rigorous revision process was undertaken, resulting in the removal of four items and the modification of five others. Items were primarily deleted based on two conditions: an average relevance rating below 3.0 or low CVI values combined with clear semantic redundancy. For example, an item concerning difficulty with efficient assignment completion was deemed duplicative of another item addressing the inability to manage complex tasks without AI. As a result, the latter was retained for its conceptual breadth. Similarly, an item describing feeling “unsettled” was removed due to its overlap with a clearer statement about feeling “uneasy or insecure”. Item wording was also refined for precision, such as revising a statement about a decline in the “ability to independently analyze” to the more comprehensive “ability to independently analyze and solve problems”. This iterative review process culminated in a revised 22-item scale. A summary of the specific item modifications based on the expert review is presented in Table 2.

**Table 2:** Summary of item modifications based on expert review.

**Action taken**	**Item ID**	**Rationale for change**	**Final wording**
Deleted	Item8	Redundant with Item10	N/A
	Item4	Overlap with Item1	N/A
	Item19	Conceptually redundant with Item17	N/A
	Item20	Overlap with Item25	N/A
Revised	Item15	Increase specificity and scope	After using AI, my ability to independently analyze and solve problems has declined.
	Item25	To improve clarity and reduce length	I am capable of completing tasks without AI, but I still cannot resist using it.
	Item11	Clarified purpose of task delegation	I prefer to delegate information gathering and content organization to AI rather than doing it entirely myself.
	Item17	Refined to reduce subjectivity	After long-term use of AI, I have become less inclined to think independently, preferring AI to do the work.
	Item23	Shortened for readability	I know I shouldn't rely too much on AI, but I can't control myself.

### 2.3 Pilot testing and cognitive interviews

Prior to large-scale data collection and analysis, a pilot study involving 30 undergraduate students was conducted to evaluate the comprehensibility and preliminary psychometric properties of the 22-item AI Dependence Scale. This mixed-methods phase integrated both quantitative and qualitative evaluations.

The quantitative component consisted of a preliminary reliability analysis. The results demonstrated strong internal consistency, with a Cronbach's alpha of 0.91 for the total scale and subscale alphas ranging from 0.85 to 0.89. Additionally, all items exhibited corrected item-total correlations substantially exceeding the 0.40 threshold, providing robust initial evidence for their retention. For the qualitative component, brief cognitive interviews were conducted with participants. They were prompted to articulate their interpretation of each item and to identify any ambiguous or confusing wording. These interviews confirmed that item interpretations were consistent with the intended constructs and that the scale was generally clear. Following these minor phrasing adjustments, the instrument was finalized as the AIDep-22 for the subsequent validation and analysis.

### 2.4 Participants and data collection

The finalized AIDep-22 was administered to two independent samples of undergraduate students to permit sequential EFA and CFA. Both samples were recruited from a comprehensive university in Southwest China. A stratified sampling strategy based on academic major was employed to ensure a diverse and comparable representation of disciplines in both samples. Specifically, all undergraduate programs at this university were grouped into four major categories: natural sciences, social sciences, arts, and education, which served as the sampling strata. Within each stratum, intact classes were randomly selected and invitation quotas were set proportionally to the overall enrollment of each major category. Data was collected anonymously from an online questionnaire platform. Links were then distributed by course instructors and shared in groups within the learning management system for two weeks.

The first sample (*N* = 400), designated for the EFA, was composed of 281 females (70.2%) and 119 males (29.8%). The participants' ages ranged from 18 to 24 years (*M* = 21.19, *SD* = 1.48). This sample included students from all 4 academic years, with the largest groups being seniors (28.7%) and juniors (28.5%). In terms of weekly AI usage, the majority of students (74.0%) reported using AI tools one to four times per week, while 15.0% reported more frequent use (five or more times per week).

The second sample (*N* = 400), reserved for the CFA and subsequent validation, was collected one month later from a distinct cohort of students at the same university, and used the same recruitment channels to maintain consistency in procedures. This sample consisted of 259 females (64.8%) and 141 males (35.2%), with an age distribution that was highly comparable to the first sample, ranging from 18 to 24 years (*M* = 21.17, *SD* = 1.51). The distribution of academic majors was similar to Sample 1, reflecting the stratified sampling design. The representation across academic years was also broadly similar, with sophomores (30.0%) and freshmen (26.8%) comprising the largest groups. A comparable pattern of AI usage was observed, with the majority of students (74.0%) using AI one to four times per week and 15.0% reporting use five or more times. A comprehensive demographic breakdown for both samples is presented in Table 3. These similarities established the second sample as a suitable group for validation analyses.

**Table 3:** Descriptive statistics of the study samples.

**Characteristic**	**Group**	**Sample 1** ***N*** = **400**		**Sample 2** ***N*** = **400**	
		***N***	**Frequency (%)**	***N***	**Frequency (%)**
Gender	Female	281	70.2	259	64.8
	Male	119	29.8	141	35.2
Age	≤ 21	230	57.5	243	60.8
	≥22	170	42.5	157	39.2
Academic year	Freshman	104	26.0	107	26.8
	Sophomore	67	16.8	71	17.8
	Junior	114	28.5	120	30.0
	Senior	115	28.7	102	25.5
Major category	Natural Sciences	160	40.0	160	40.0
	Social Sciences	130	32.5	130	32.5
	Arts	67	16.8	67	16.8
	Education	43	10.8	43	10.8
Usage of AI (per week)	Never/Rarely	44	11.0	44	11.0
	1–2 times	172	43.0	180	45.0
	3–4 times	124	31.0	116	29.0
	≥5 times	60	15.0	60	15.0

The study protocol received approval from the Institutional Review Board (IRB) of the researchers' university. All participants were provided with a detailed information sheet and gave written informed consent prior to participation. The data collection process was voluntary and anonymous. Participants were fully informed of the study's purpose, the exclusive use of their data for research, and their right to withdraw at any time without penalty. All procedures were conducted in accordance with the ethical standards stipulated in the Declaration of Helsinki.

## 3 Data analysis

This section details the comprehensive psychometric evaluation of the AIDep-22. The primary objective is to establish the scale's structural validity, reliability, and criterion validity through a sequential, multi-stage analytical approach. All statistical analyses were performed using IBM SPSS 27.0 for the EFA and reliability tests, while AMOS 27.0 was utilized for the CFA.

### 3.1 Exploratory factor analysis

**Figure 2:**
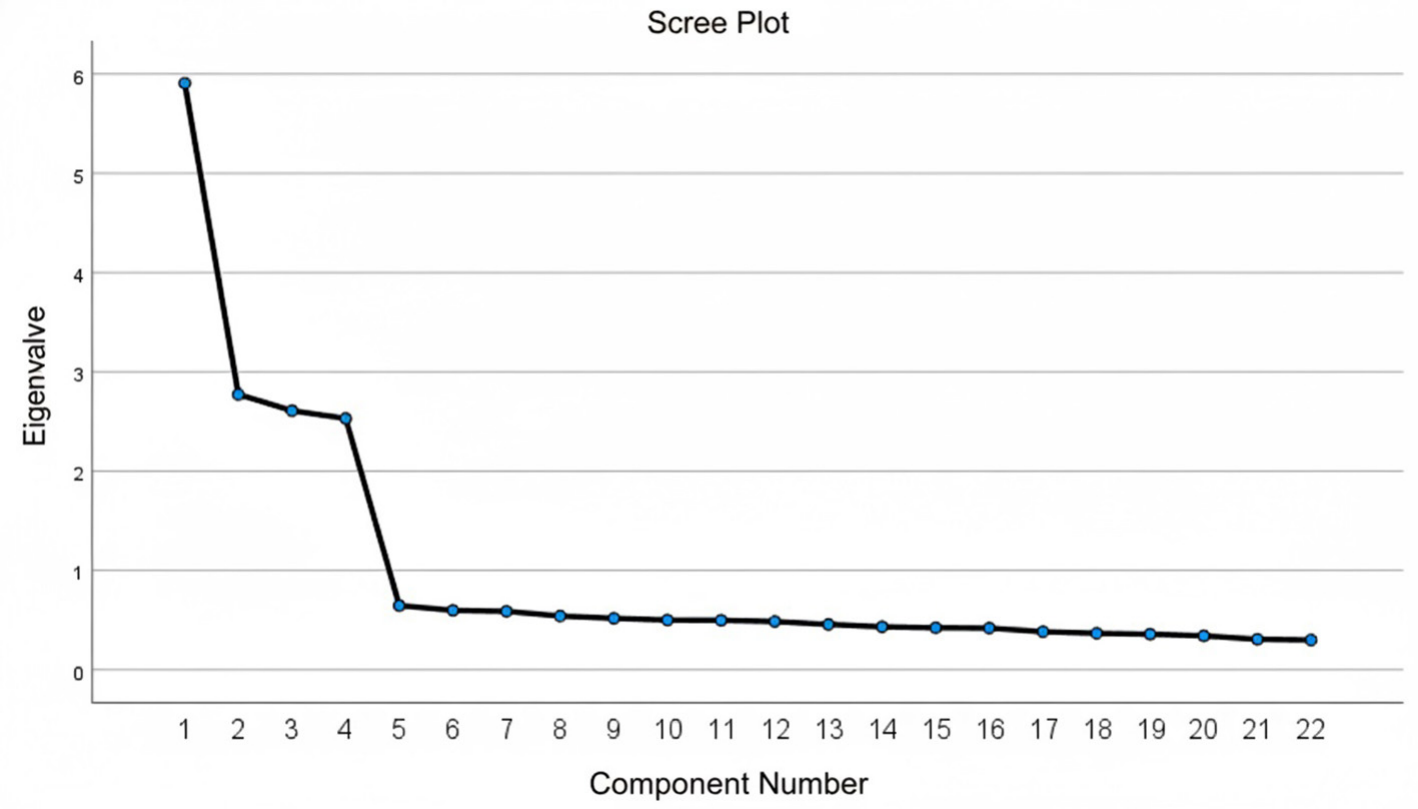
The scree plot from the EFA.

The EFA was conducted on data from Sample 1 to identify the underlying factor structure of the AIDep-22. Prior to extraction, the suitability of the data for factor analysis was confirmed. The Kaiser-Meyer-Olkin (KMO) measure of sampling adequacy was 0.893, well above the recommended value of 0.60, and Bartlett's test of sphericity was significant, χ^2^(231) = 3876.32, *p* < 0.001, indicating that the correlation matrix was suitable for factorization (Hooper, 2012). All items also exhibited satisfactory communalities, ranging from 0.57 to 0.71, indicating that a substantial proportion of each item's variance was accounted for by the extracted factors. The scree plot (Figure 2) revealed a clear elbow after the fourth factor, further supporting the retention of a four-factor solution.

Principal component analysis with a Varimax orthogonal rotation was then employed for factor extraction, using Kaiser's criterion of retaining factors with eigenvalues greater than one (Kaiser, 1960). A Varimax rotation was chosen instead of an oblimin rotation because the four dimensions were conceptualized as distinct first-order constructs, and the primary goal at this exploratory stage was to obtain a clear interpretable simple structure, while factor inter-correlations were examined more rigorously in the subsequent CFA. This procedure yielded a four-factor solution that aligned with the proposed theoretical model. Collectively, these four factors accounted for 62.78% of the total variance, exceeding the conventional 60% threshold for satisfactory explanatory power. Standard retention criteria were applied: a primary factor loading of 0.50 or higher, no cross-loadings exceeding 0.35, and a minimum of three items per factor. All 22 items met these criteria, necessitating no further deletions. As shown in Table 4, each item loaded strongly onto a single intended factor, with negligible loadings on other factors, supporting a clean and well-defined factor structure. The variance explained by each factor was as follows: Factor 2 (17.36%), Factor 4 (16.41%), Factor 3 (14.65%), and Factor 1 (14.36%).

**Table 4:** Exploratory factor analysis and rotated factor loadings of the AIDep-22 (sample 1).

**Item code**	**Emotional dep**	**Functional dep**	**Cognitive dep**	**Loss of control**	**Communalities**
Item1	0.802				0.652
Item2	0.782				0.637
Item3	0.755				0.602
Item5	0.763				0.616
Item6	0.782				0.625
Item7		0.766			0.604
Item9		0.768			0.614
Item10		0.773			0.633
Item11		0.814			0.705
Item12		0.788			0.640
Item13		0.791			0.671
Item14			0.790		0.644
Item15			0.784		0.638
Item16			0.790		0.639
Item17			0.771		0.631
Item18			0.791		0.659
Item21				0.755	0.591
Item22				0.765	0.600
Item23				0.735	0.581
Item24				0.792	0.642
Item25				0.747	0.574
Item26				0.759	0.622

Dep, Dependence. KMO =0.893; Bartlett's Test of Sphericity: χ^2^(231) = 3876.32.

*p* < 0.0001. Factor loadings below.40 are not shown.

The EFA results confirmed that the AIDep-22 captures four distinct dimensions of AI dependence, corresponding directly to the study's theoretical constructs. The emergent factors were clearly interpretable and thematically aligned with their respective item content. In contrast, a forced single-factor solution demonstrated poorer fit, explaining only approximately 27% of the variance, confirming the inadequacy of a unidimensional model. Following the EFA, internal consistency was assessed. Cronbach's alpha for the AIDep-22 was 0.867. The subscales also demonstrated high reliability: emotional dependence (α = 0.849), functional dependence (α = 0.886), cognitive dependence (α = 0.858), and loss of control (α = 0.865).

### 3.2 Confirmatory factor analysis

A CFA was performed on the Sample 2 dataset to validate the four-factor structure identified during the exploratory phase. The measurement model specified four first-order latent constructs: emotional dependence, functional dependence, cognitive dependence, and loss of control, each representing a distinct dimension of AI dependence. These four factors were further modeled as indicators of a higher-order latent construct representing overall AI dependence.

**Figure 3:**
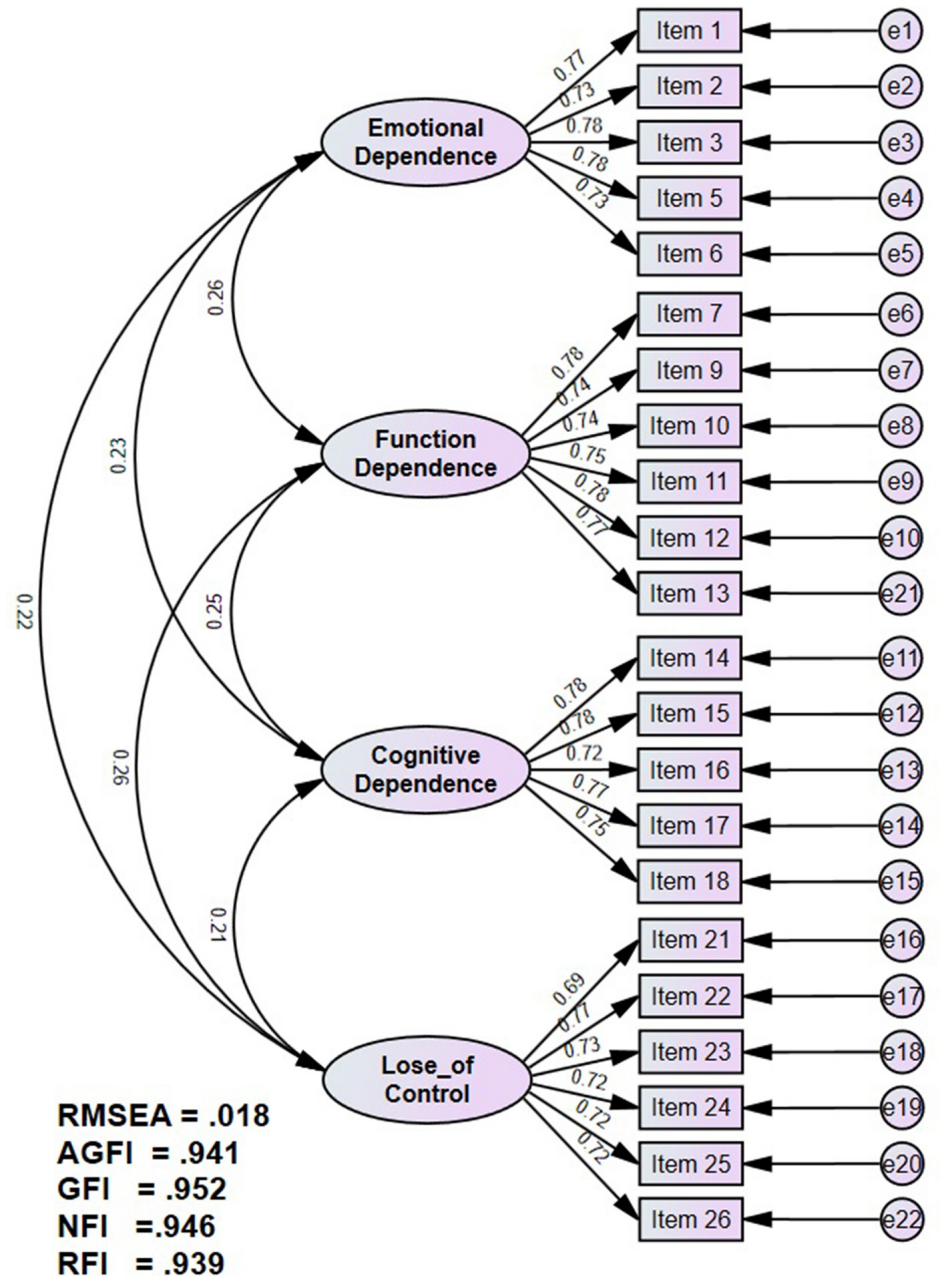
The CFA model of this study.

Model fit was assessed using multiple indices, including the chi-square statistic, the ratio of chi-square to degrees of freedom (χ^2^/df), the Comparative Fit Index (CFI), the Tucker-Lewis Index (TLI), the Root Mean Square Error of Approximation (RMSEA), and the Standardized Root Mean Square Residual (SRMR). The final model is presented in Figure 3. The hypothesized four-factor model demonstrated an adequate fit to the data: χ^2^ (203) = 228.885, p =0.103; χ^2^/df = 1.128; CFI = 0.994; TLI = 0.993; RMSEA = 0.018, 90% CI [0.000, 0.029]; and SRMR = 0.052. The low RMSEA value and its narrow confidence interval, together with the small SRMR, indicate a close fit of the model to the data. The chi-square test was non-significant, and the other indices all met conventional thresholds (Hu and Bentler, 1999). Competing models, including a one-factor solution and models with fewer than four factors, exhibited substantially poorer fit, further confirming the superiority of the four-factor specification. Modification indices were inspected and did not reveal any theoretically meaningful misspecifications; therefore, no *post-hoc* model modifications were applied and the original four-factor structure was retained.

As presented in Table 5, all items loaded significantly on their intended factors (*p* < 0.001), with standardized factor loadings ranging from 0.69 to 0.78. Each factor displayed strong internal consistency, with composite reliability (CR) values ranging from 0.87 to 0.89. The average variance extracted (AVE) ranged from 0.53 to 0.58, surpassing the recommended threshold of 0.50, thereby supporting convergent validity (Fornell and Larcker, 1981).

**Table 5:** Confirmatory factor analysis of the AIDep-22 (sample2).

**Dimensions and items**	**Loading**	**CR**	**AVE**
**Emotional dependence**		0.87	0.58
Item1: I feel uneasy or insecure without the help of AI.	0.767^***^		
Item2: Using AI to assist my studies makes me feel calm and relaxed.	0.730^***^		
Item3: I would feel anxious and frustrated if I couldn't use AI for my studies.	0.784^***^		
Item5: Whenever I encounter a difficult problem, my first instinct is to hope AI can help me; otherwise, I feel tense.	0.780^***^		
Item6: Interacting with AI via chat prompts is a pleasant experience that makes me feel supported.	0.730^***^		
**Functional dependence**		0.89	0.58
Item7: I delegate almost all my academic tasks to AI because it performs them faster and better than I do.	0.775^***^		
Item9: I rely on AI's functions, including providing answers and writing suggestions, to improve my learning efficiency.	0.741^***^		
Item10: I can no longer manage complex academic tasks without the assistance of AI.	0.739^***^		
Item11: I prefer to delegate information gathering and content organization to AI rather than doing it entirely myself.	0.751^***^		
Item12: In my studies, I excessively rely on the ready-made answers or solutions provided by AI.	0.783^***^		
Item13: I feel inadequate in my studies without the support of AI.	0.765^***^		
**Cognitive dependence**		0.87	0.58
Item14: I often ask AI for answers directly without thinking for myself first.	0.776^***^		
Item15: After using AI, my ability to independently analyze and solve problems has declined.	0.775^***^		
Item16: When faced with an academic challenge, I rely on AI instead of attempting to think it through on my own.	0.723^***^		
Item17: After long-term use of AI, I have become less inclined to think independently, preferring AI to do the work.	0.773^***^		
Item18: I am accustomed to letting AI make judgments and decisions for me rather than fully trusting my own judgment.	0.753^***^		
**Loss of control**		0.87	0.53
Item21: I have tried to reduce my use of AI, but I have never succeeded for long.	0.693^***^		
Item22: Once I start using AI, I often find it difficult to stop, even after spending a lot of time on it.	0.768^***^		
Item23: I know I shouldn't rely too much on AI, but I can't control myself.	0.727^***^		
Item24: I have almost no control over the frequency and duration of my AI use.	0.720^***^		
Item25: I am capable of completing tasks without AI, but I still cannot resist using it.	0.716^***^		
Item26: If I don't use AI for a period of time, I feel a strong urge to use it, sometimes even feeling irritable.	0.719^***^		

*N* = 400. All factor loadings are standardized. CR, Composite Reliability; AVE, Average Variance Extracted.

^***^p < 0.001.

### 3.3 Convergent and discriminant validity

The convergent validity of the AIDep-22 was demonstrated through multiple indicators. As reported in Table 5, all standardized item loadings were statistically significant, ranging from 0.69 to 0.78, which is generally acceptable for scale development. Furthermore, the CR values for the four dimensions ranged from 0.87 to 0.89, and the AVE values were between 0.53 and 0.58. These results confirm that each construct explains more than half of the variance in its associated items, thereby providing strong evidence of convergent validity.

Discriminant validity was evaluated using the Fornell-Larcker criterion. As presented in Table 6, the square root of the AVE for each construct (bold diagonal entries) was greater than its correlation with any other construct. For instance, the square root of the AVE for emotional dependence was 0.76, which exceeded its highest inter-factor correlation (*r* = 0.26). The inter-factor correlations ranged from a low of 0.21 to a high of 0.26, indicating that the four dimensions are moderately related yet empirically distinct. This provides compelling evidence for the discriminant validity of the AIDep-22.

**Table 6:** Discriminant validity based on the Fornell–Larcker criterion of the AIDep-22(sample 2).

**Dimension**	**Emotional dep**	**Functional dep**	**Cognitive dep**	**Loss of control**
Emotional dep	0.76			
Functional dep	0.26	0.76		
Cognitive dep	0.23	0.25	0.76	
Loss of control	0.22	0.26	0.21	0.73

Dep, Dependence. Diagonal elements represent the square roots of the AVE; off-diagonal elements are the correlations between factors. All correlations are significant at *p* < 0.001.

### 3.4 Criterion validity

Criterion validity of the AIDep-22 was assessed against the validated Academic Self-Efficacy Scale (Pribeanu et al., 2023) and behavioral indicators of AI use derived from Burton-Jones and Straub's (2006) conceptual framework of system usage. Effect sizes are interpreted using Cohen's (1988) conventions.

As shown in Table 7, AIDep-22 was significantly and negatively correlated with ASE. The total dependence score exhibited a medium-to-large negative correlation with total ASE (*r* = −0.46), underscoring an inverse relationship between AI dependence and academic confidence. The negative associations were most pronounced for self-efficacy with self-regulated learning (SEA, *r* = −0.35) and self-efficacy with the course (SEC, *r* = −0.32), suggesting that AI dependence is more closely tied to students' confidence in their core academic and self-regulatory abilities rather than their social or general technical skills.

**Table 7:** Correlation between AIDep-22 and criterion factors (sample 2).

**Scale**	**Total**	**Emotional**	**Functional**	**Cognitive**	**Loss of control**
ASE total	−0.46^*^	−0.32^*^	−0.28^*^	−0.29^*^	−0.28^*^
SEA	−0.35^*^	−0.25^*^	−0.17^*^	−0.25^*^	−0.22^*^
SEC	−0.32^*^	−0.24^*^	−0.23^*^	−0.20^*^	−0.14^*^
SSE	−0.19^*^	−0.10^*^	−0.13^*^	−0.13^*^	−0.14^*^
CSE	−0.15^*^	−0.12^*^	−0.09+	−0.06	−0.11^*^
Extent of use	0.33^*^	0.22^*^	0.34^*^	0.15^*^	0.14^*^
Variety of use	0.48^*^	0.31^*^	0.40^*^	0.29^*^	0.22^*^
Frequency of use	0.37^*^	0.22^*^	0.30^*^	0.25^*^	0.16^*^
Proportion of use	0.30^*^	0.14^*^	0.27^*^	0.18^*^	0.17^*^
Duration of use	0.12^*^	0.04	0.09+	0.10^*^	0.08

*N* = 400. ASE, Academic Self-Efficacy; SEA, Self-efficacy with Self-regulated learning; SEC, Self-efficacy with the Course; SS, Social Self-efficacy; CSE, Computer Self-efficacy.

^*^p < 0.05; +*p* < 0.10.

In contrast, strong positive associations were observed between AIDep-22 and behavioral usage. Higher dependence was most strongly associated with a greater variety of use (*r* = 0.48), followed by frequency (*r* = 0.37) and extent of use (*r* = 0.33). The association with duration of use (*r* = 0.12) was significantly weaker, indicating that the sheer amount of time spent is a less telling measure of psychological dependence than the breadth and frequency of use.

Multiple regression analyses further substantiated these associations as shown in Table 8. All four dependence dimensions collectively explained a significant portion of the variance in ASE (*R*^2^ = 0.214), with each dimension emerging as a significant, independent negative predictor. This demonstrates that AI dependence is a multifaceted construct that undermines students' confidence. These results are consistent with prior findings showing that reliance on AI tools undermines students' perceived academic ability (Hou et al., 2025; Zhai et al., 2024). Regarding behavior, functional dependence was the most powerful predictor of use, particularly for variety (β = 0.30) and extent (β = 0.29), suggesting that the perceived necessity of AI for task completion is a primary driver of its widespread application. In conclusion, these results provide strong evidence for the criterion-related validity of the AIDep-22.

**Table 8:** Summary of multiple regression analyses for criterion validity (sample 2).

**Scale**	**ASE total**			**SEA**			**SEC**			**SSE**			**CSE**		
	β	***R*** ^2^	***p***	β	***R*** ^2^	***p***	β	***R*** ^2^	***p***	β	***R*** ^2^	***p***	β	***R*** ^2^	***p***
Emotional	−0.22		< 0.001^*^	−0.17		< 0.001^*^	−0.18		0.003^*^	−0.05		0.126	−0.09		0.082+
Functional	−0.16		0.021^*^	−0.06		0.148	−0.15		0.018^*^	−0.08		0.162	−0.05		0.238
Cognitive	−0.18		0.006^*^	−0.18		0.008^*^	−0.12		0.031^*^	−0.08		0.118	−0.02		0.352
Loss of control	−0.17		0.011^*^	−0.14		0.024^*^	−0.05		0.186	−0.10		0.142	−0.08		0.195
AIDep-22		0.21	< 0.001^*^		0.13	< 0.001^*^		0.11	< 0.001^*^		0.04	< 0.001^*^		0.03	< 0.001^*^
**Scale**	**Extent of use**			**Variety of use**			**Frequency of use**			**Proportion of use**			**Duration of use**		
	β	***R*** ^2^	***p***	β	***R*** ^2^	***p***	β	***R*** ^2^	***p***	β	***R*** ^2^	***p***	β	***R*** ^2^	***p***
Emotional	0.13		0.034^*^	0.19		0.011^*^	0.13		0.072+	0.05		0.182	0.00		0.412
Functional	0.29		< 0.001^*^	0.30		< 0.001^*^	0.23		< 0.001^*^	0.22		0.009^*^	0.06		0.164
Cognitive	0.06		0.091+	0.17		0.032^*^	0.16		0.041^*^	0.10		0.104	0.07		0.214
Loss of control	0.04		0.152	0.08		0.095+	0.05		0.188	0.09		0.138	0.05		0.216
AIDep-22		0.140	< 0.001^*^		0.246	< 0.001^*^		0.145	< 0.001^*^		0.101	< 0.001^*^		0.018	0.019^*^

*N* = 400. β, standardized coefficient. ASE, Academic Self-Efficacy (SEA, Self-efficacy with Self-regulated learning; SEC, Self-efficacy with the Course; SSE, Social Self-efficacy; CSE, Computer Self-efficacy).

^*^p < 0.05; +*p* < 0.10.

## 4 Preliminary exploration: demographic differences in AI dependence

In addition to validating the psychometric properties of the AIDep-22, this study investigated the associations between key demographic factors and AI dependence among Chinese undergraduates. Such an analysis is crucial for identifying student cohorts at an elevated risk of problematic reliance. The investigation centered on five key variables: gender, age, academic year, major category, and frequency of AI use. To ensure sufficient statistical power, the analyses were conducted on a combined dataset (N = 800), an approach validated by the established configural and metric invariance across the two samples in the EFA and CFA.

Table 9 presents the descriptive statistics for AI dependence and its four dimensions among the participants. All subscales showed good internal consistency reliability, with Cronbach's alpha ranging from 0.86 to 0.89, and the total scale α = 0.87. Skewness and kurtosis values indicated acceptable univariate normality. These descriptive results indicate that AI dependence among participants was at a moderate-to-high level (*M* = 3.94, SD = 0.39). Functional dependence had the highest mean score (*M* = 4.08), suggesting that students primarily rely on AI for academic task completion, while loss of control showed the lowest mean (*M* = 3.87), reflecting a relatively low yet still notable difficulty in regulating AI use.

**Table 9:** Descriptive statistics (combine sample 1 and sample 2).

**Scale**	**Min**	**Max**	**M**	**SD**	**Skew**	**Kurtosis**	**Cronbach's alpha**
Emotional dep	1.80	5.00	3.90	0.64	−0.27	−0.17	0.86
Functional dep	1.67	5.00	4.08	0.60	−0.46	0.53	0.89
Cognitive dep	1.80	5.00	3.91	0.64	−0.20	−0.19	0.87
Loss of control	1.83	5.00	3.87	0.55	−0.24	0.54	0.87
Total	2.41	5.00	3.94	0.39	−0.00	1.32	0.87

*N* = 800; M, Mean; SD, Standard Deviation; Dep, Dependence.

### 4.1 Gender and age

An independent-samples t-test revealed a significant gender difference in AI dependence, with male students (*M* = 4.01) reporting higher levels than female students (*M* = 3.91). As shown in Table 10, this disparity is not uniform. The results demonstrate that males scored significantly higher, specifically on emotional dependence (*M* = 4.09 vs. 3.81, *p* < 0.001) and cognitive dependence (*M* = 4.03 vs. 3.85, *p* < 0.001). Conversely, scores for functional dependence and loss of control showed no significant gender differences. This specific pattern suggests that the higher AI dependence observed in male students is primarily rooted in their greater reliance on AI for emotional regulation and as a substitute for their own cognitive efforts, rather than only for practical task completion. This finding can be contextualized by existing research indicating that men often report more positive attitudes and higher confidence in using technology (Møgelvang et al., 2024). However, it is crucial to distinguish this psychological dependence from actual competence. For instance, (Mashburn et al. 2025) found that while female medical professionals initially rated their expertise lower, they demonstrated significantly greater knowledge improvement after using ChatGPT compared to their male counterparts. This underscores the unique value of the AIDep-22 in identifying a psychological vulnerability that operates independently of performance.

**Table 10:** Independent-samples t-tests by gender and age.

**Dimensions**	**Factor**	**Group1**	**Group2**	**M1**	**M2**	**MD**	**t**	***p***	**d**
Emotional dep	Gender	Male	Female	4.09	3.81	0.28	5.91	< 0.001^***^	0.441
Functional dep				4.08	4.08	0.00	0.07	0.948	0.005
Cognitive dep				4.03	3.85	0.18	3.87	< 0.001^***^	0.279
Loss of control				3.86	3.87	−0.01	−0.16	0.877	−0.012
AIDep-22				4.01	3.91	0.10	3.48	< 0.001^***^	0.265
Emotional dep	Age	≤ 21	≥22	3.86	3.96	−0.10	−2.12	0.035^*^	−0.153
Functional dep				4.00	4.19	−0.19	−4.52	< 0.001^***^	−0.320
Cognitive dep				3.86	3.98	−0.12	−2.63	0.009^**^	−0.189
Loss of control				3.85	3.89	−0.05	−1.18	0.240	−0.084
AIDep-22				3.89	4.01	−0.11	−4.11	< 0.001^***^	−0.295

*N* = 800; M, Mean; MD, Mean Difference; d, Cohen's d; AIDep-22, AI Dependence Scale; Dep, Dependence. ^*^p < 0.05, ^**^p < 0.01, ^***^p < 0.001.

Table 10 also indicates that older students (≥ 22 years, *M* = 4.01) report significantly higher AI dependence than their younger peers ( ≤ 21 years, *M* = 3.89). This effect is consistent across multiple dimensions, with significant differences observed in functional (MD = 0.19), cognitive (MD = 0.12), and emotional dependence (MD = 0.10). Although previous research has frequently characterized younger individuals as the primary adopters and frequent users of generative AI technologies in educational and writing contexts (Higgs and Stornaiuolo, 2024), emerging empirical evidence suggests that such reliance is not confined to the youngest cohorts. For instance, (Baek et al. 2024) reported that older university students, particularly those aged between 30 and 40 years, were more likely to use ChatGPT on a frequent basis than their younger counterparts. This growing body of evidence suggests that age-related differences in AI engagement may follow a more complex and developmental trajectory rather than a simple generational divide. In this context, the present finding that older undergraduates demonstrate greater AI dependence across emotional, functional, and cognitive dimensions indicates that AI reliance may intensify with increasing academic experience and task complexity. This trend will be further examined in the subsequent analysis by academic year.

### 4.2 Academic year

Expanding on the age-related findings, the one-way analysis of variance (ANOVA) by academic year detailed the evidence of how AI dependence progresses. Table 11 shows a statistically significant and strong effect of academic year on AI dependence (*F* (3, 796) = 13.05, *p* < 0.001). The partial eta-squared value (ηp^2^ = 0.047) indicates that academic year accounts for a meaningful portion (4.7%) of the variance in dependence scores, highlighting its practical significance. Notably, functional dependence exhibits the largest effect size among the dimensions (ηp^2^ = 0.041). This specific pattern provides a powerful insight: as students advance academically, the primary change is a dramatic increase in their dependence on AI as a functional tool to complete academic tasks. While dependence in other dimensions also grows, it is the instrumental, task-oriented usage that defines the escalating reliance in later academic years. This functionally driven progression offers a more nuanced explanation for the initial t-test result where older students showed higher dependence. This finding solidifies the conclusion that AI dependence is a developmental issue within the university context, reinforcing previous research on conversational AI (Chen et al., 2025) and identifying upper-year students as a key at-risk group facing increasing academic complexity and pressure.

**Table 11:** One-way ANOVA by academic year.

**Dimension**	**Levene F**	**Levene p**	**Between SS**	**df**	**MS**	***F***	***p***	**ηp^2^**	**Bonferroni**
Emotional dep	0.46	0.713	3.30	3	1.10	2.68	0.046^*^	0.010	1 < 2 < 3 < 4
Functional dep	0.59	0.624	11.58	3	3.86	11.22	< 0.001^***^	0.041	1 < 2 < 3 < 4
Cognitive dep	0.43	0.730	5.79	3	1.93	4.74	0.003^**^	0.018	1 < 2 < 3 < 4
Loss of control	1.47	0.223	4.65	3	1.55	5.24	0.001^**^	0.019	1 < 2 < 3 < 4
Total	0.27	0.847	5.69	3	1.90	13.05	< 0.001^***^	0.047	1 < 2 < 3 < 4

*N* = 800; SS, Sum of Squares; df, degrees of freedom; MS, Mean Square; ηp^2^, partial eta-squared; Dep, Dependence. In the Bonferroni column, numbers represent academic years (1–4).^*^p < 0.05, ^**^p < 0.01, ^***^p < 0.001.

### 4.3 Major Category

The analysis reveals that major category is another significant factor influencing the AI dependence of undergraduates (*F* (3, 796) = 7.59, *p* < 0.001). As shown in Table 12, this overall effect is primarily driven by significant differences in emotional dependence (*F* = 8.23, *p* < 0.001) and, most notably, functional dependence (*F* = 20.76, *p* < 0.001), which has a large effect size (ηp^2^ = 0.073). The Bonferroni *post-hoc* comparisons indicate a consistent hierarchy, with education majors reporting the highest dependence, followed by arts, social sciences, and finally natural sciences. The disciplines in this study with a strong practical or “applied” orientation (e.g., education) exhibit the highest levels of dependence, particularly functional dependence. This strongly suggests that the problem-solving and task-oriented nature inherent in applied fields fosters greater reliance on AI as a practical tool. This interpretation aligns with the findings of (Qu et al. 2024), who observed that students in applied fields not only possess greater AI knowledge but also engage more with AI for complex cognitive tasks. The AIDep-2′s ability to pinpoint functional dependence as the primary driver of these disciplinary differences showcases its value in understanding how and why different academic cultures cultivate distinct patterns of AI dependence.

**Table 12:** One-way ANOVA by major category.

**Dimension**	**Levene F**	**Levene p**	**Between SS**	**df**	**MS**	***F***	***p***	**ηp^2^**	**Bonferroni**
Emotional dep	1.83	0.140	9.93	3	3.31	8.23	< 0.001^***^	0.030	NatSci < SocSci < Arts < Edu
Functional dep	0.97	0.406	20.71	3	6.90	20.76	< 0.001^***^	0.073	NatSci < SocSci < Arts < Edu
Cognitive dep	2.94	0.032	–	–	–	–	–	–	–
Loss of control	0.60	0.614	0.07	3	0.02	0.08	0.973	0.000	–
Total	0.84	0.473	3.37	3	1.12	7.59	< 0.001^***^	0.028	NatSci < SocSci < Arts < Edu

*N* = 800; SS, Sum of Squares; df, degrees of freedom; MS, Mean Square; ηp^2^, partial eta-squared; NatSci, Natural Sciences; SocSci, Social Sciences; Edu, Education; Dep, Dependence.“–” indicates that the F-test was not conducted due to violation of the homogeneity of variances assumption or was not significant.

^***^p < 0.001.

### 4.4 AI usage frequency

As shown in Table 13, the analysis confirms a very strong, positive relationship between the frequency of AI use and the level of overall AI dependence (*F* (3, 796) = 27.95, *p* < 0.001), with a large effect size (ηp^2^ = 0.095). This result provides robust known-groups validity for the AIDep-22. The Bonferroni *post-hoc* test for the total score clarifies this with a clear dose-response relationship, showing that dependence progressively increases with each rise in usage frequency, from “Never/Rarely” through “≥ 5 times per week”.

**Table 13:** One-way ANOVA by AI usage frequency (*N* = 800).

**Dimension**	**Levene F**	**Levene p**	**Between SS**	**df**	**MS**	***F***	***p***	**ηp^2^**	**Bonferroni**
Emotional dep	1.39	0.244	8.03	3	2.68	6.61	< 0.001^***^	0.024	1 < 4 < 2 < 3
Functional dep	4.24	0.005	–	–	–	–	–	–	–
Cognitive dep	1.77	0.151	12.00	3	4.00	10.02	< 0.001^***^	0.036	1 < 2 < 4 < 3
Loss of control	4.65	0.003	–	–	–	–	–	–	–
Total	1.86	0.135	11.56	3	3.85	27.95	< 0.001^***^	0.095	1 < 2 < 3 < 4

*N* = 800; SS, Sum of Squares; df, degrees of freedom; MS, Mean Square; Dep, Dependence; ηp^2^, partial eta-squared. Per week AI usage: 1, Never/Rarely; 2, 1–2 times; 3, 3–4 times; 4, ≥5 times. “–” indicates the F-test was not conducted due to violation of the homogeneity of variances assumption.

^***^p < 0.001.

However, a more nuanced pattern emerges from the subscales. The developmental trajectory of dependence is not uniformly linear. For emotional dependence and cognitive dependence, the *post-hoc* tests reveal a non-linear trend. Dependence in these two areas peaks not among the most frequent users (≥ 5 times per week), but among the moderate-to-heavy users (3–4 times per week). This suggests that the initial stages of increasing AI engagement are the most critical period for developing psychological and cognitive reliance. The heaviest users, while still highly dependent, may transition to a more instrumental mode of use, a phenomenon that could indicate a “habituation” effect.

It is important to note that a definitive *post-hoc* analysis for functional dependence and loss of control was precluded by a violation of the homogeneity of variances assumption. Nonetheless, the available data strongly suggest that the journey into AI dependence is complex. This is consistent with a feedback loop model in which academic struggles drive AI use (Zhang et al., 2024). These findings add a crucial detail: the psychological and cognitive aspects of this dependence appear most acute during the ramp-up phase of usage. This highlights the importance of early intervention for students who are beginning to intensify their reliance on AI tools.

## 5 Discussion

The results of this study demonstrate that AI dependence presents a clear multidimensional structure and a differentiated distribution across the undergraduates. This section provides an in-depth discussion and analysis of the theoretical and practical implications of these findings.

### 5.1 Theoretical implications

The findings of this study validate the AIDep-22 as a robust instrument and offer significant theoretical contributions by empirically confirming the multidimensional framework. By integrating behavioral addiction theory, technology acceptance models, and cognitive offloading perspectives, the results clarify how AI dependence operates distinctively within higher education.

First, the dominance of functional dependence offers a critical correction to traditional TAM in academic contexts. While TAM posits that perceived usefulness drives positive adoption, the high scores observed in this dimension, particularly among senior students and applied majors, suggest a theoretical turning point where usefulness mutates into dependency. This aligns with recent theoretical arguments that reliance creates a feedback loop of perceived inadequacy where the system becomes indispensable (Parveen et al., 2024; Shahzad et al., 2024). The data supports this by showing that as academic tasks become more complex for older students, the instrumental efficiency of AI evolves from a convenience into a psychological necessity. This extends the arguments of (Zhai et al. 2024) by demonstrating that in academic environments, high functional value is paradoxically the primary precursor to autonomy loss rather than merely a marker of successful technology integration.

Second, the findings regarding cognitive dependence provide empirical weight to the cognitive offloading framework. This resonates with the premise that delegating higher order thinking to external tools systematically displaces internal processing and results in skill atrophy (Grinschgl et al., 2021; Gilbert, 2024). This study confirms this through the negative association between cognitive dependence and academic self-efficacy. Notably, the peak of this dependence among moderate frequency users challenges the assumption that only heavy users are at risk (Baek et al., 2024). It suggests that the “illusion of competence” described by (Gonsalves 2024) establishes itself early in the adoption curve. This indicates that cognitive offloading theory should be expanded to address the qualitative displacement of thinking processes even when usage frequency remains moderate.

Third, the emotional dependence results refine the application of behavioral addiction theory to AI. Unlike social media addiction which is driven by interpersonal fear of missing out, the gender differences identified in this study point to a different mechanism. Male students exhibited significantly higher emotional and cognitive dependence. This supports the notion that AI functions as a “safe space” for academic vulnerability as noted by (Hu et al. 2023). The results suggest that for male students, who often report higher technical confidence, the AI serves as a private and non-judgmental buffer against academic anxiety. This shifts the theoretical lens from social validation to performance anxiety regulation, and highlights that in higher education emotional dependence on AI is rooted in the solitary struggle with academic standards.

Fourth, the Loss of control dimension validates the distinction between heavy engagement and compulsive use. This corresponds with the framing of addiction as a regulatory failure where usage persists despite conflicting intentions (Griffiths, 2005; Andreassen et al., 2012). This study confirms that while functionally dependent students use AI frequently to get work done, those scoring high on loss of control struggle with the inability to disengage. This aligns with the “compulsive prompt refining” phenomena observed by (Ivanov et al. 2024). It suggests that within the academic domain, loss of control is less about the dopamine rush of content consumption and more about the perfectionistic or anxious inability to finalize tasks without algorithmic validation.

In summary, this study theoretically repositions AI dependence not merely as a habit but as a structural transformation of academic agency. It demonstrates that the mechanisms of dependence are driven primarily by the functional and cognitive outsourcing of learning demands rather than simple instant gratification. Conceptually, the robust negative associations between AI dependence and academic self-efficacy observed in this study are compatible with a potential mediation perspective, in which functional and cognitive dependence partially transmit the effects of perceived usefulness and effortless, AI-supported task completion to lower self-efficacy over time.

### 5.2 Practical implications

The multidimensional structure of the AIDep-22 model necessitates systemic pedagogical reform to effectively counter the functional and cognitive mechanisms that drive AI dependence.

To address the prominent issue of functional dependence, institutions need to redesign their assessments. The focus should shift from the final output to the learning process itself, thereby reducing the perceived need for AI to complete tasks. This involves implementing process-oriented evaluations, such as staged submissions or in-class methods, which compel students toward independent execution and strategic task planning. Meanwhile, to combat cognitive atrophy, curriculums must emphasize “human in the loop” workflows. Training needs to move beyond mere technical skills to cultivate reflective practice and critical reasoning, ensuring students utilize AI as a collaborator rather than a delegate for higher-order thinking tasks.

The study's findings on user frequency and demographics dictate the critical timing and specificity of interventions. Since both emotional and cognitive risks emerge among moderate users, mandatory preventative AI literacy training must be introduced in the early stages of a student's academic career. This training should focus on meta-cognitive awareness and responsible self-regulation (Throuvala et al., 2021). Furthermore, support must be differentiated. For male students, interventions should target academic vulnerability and anxiety reduction to mitigate the use of AI as a psychological buffer (Hu et al., 2023). Most critically, the high dependence observed among education majors requires specific ethical and pedagogical training. This is vital, as their usage habits will directly shape future school-based digital literacy practices (Stöhr et al., 2024).

### 5.3 Limitations and future research

While the present study provides robust evidence for the reliability and validity of the AIDep-22, several limitations should be acknowledged. First, the data were collected from a single university in Southwest China, a limitation that may restrict the generalizability of the results to other institutional or cultural contexts. Future research should replicate this study into multi-site and cross-cultural samples to test the stability of the factor structure and demographic patterns across diverse educational systems. Second, the study relied on self-reported measures, which are inherently subject to social desirability and recall biases. Future work should incorporate objective behavioral data, such as log data from AI platforms, to triangulate the self-reported dependence scores more objectively. Third, the study's cross-sectional design precludes causal inferences. Consequently, longitudinal designs are needed to examine the reciprocal dynamics between AI usage and the development of dependence over time.

Finally, while the AIDep-22 demonstrated strong criterion validity with academic self-efficacy and behavioral indices, further research should explore its associations with a broader range of psychological and academic outcomes, such as creativity, academic integrity, and self-regulated learning. It would also be valuable to conduct experimental studies investigating whether interventions focused on AI literacy and critical thinking can effectively mitigate the dimensions of dependence measured by the scale.

### 5.4 Conclusion

This study developed and validated the AI Dependence Scale: AIDep-22, offering a reliable multidimensional tool to measure overreliance on AI among Chinese undergraduates. By confirming four dimensions: emotional, functional, cognitive, and loss of control, the scale advances understanding of how AI integration can erode academic autonomy, extending theories of behavioral addiction and cognitive offloading. Demographic insights highlight vulnerabilities in male students, seniors, applied majors, and frequent users, informing targeted risk assessment.

Practically, the AIDep-22 empowers educators to diagnose issues early and foster interventions that emphasize process-based learning and critical AI literacy, ultimately promoting ethical technology use. Future efforts should extend validation cross-culturally, incorporate longitudinal designs, and integrate objective metrics to deepen insights into mitigating AI's potential drawbacks while harnessing its benefits for higher education.

## Data Availability

The raw data supporting the conclusions of this article will be made available by the authors, without undue reservation.
